# Lifestyle counseling during pregnancy and offspring weight development until four years of age: follow-up study of a controlled trial

**DOI:** 10.1186/1477-5751-11-11

**Published:** 2012-05-08

**Authors:** Taina Mustila, Jani Raitanen, Päivi Keskinen, Antti Saari, Riitta Luoto

**Affiliations:** 1 Central Hospital of Vaasa, Hietalahdenkatu 2 – 4, 65130, Vaasa, Finland; 2 Central Hospital of Seinäjoki, Hanneksenrinne 7, 60220, Seinäjoki, Finland; 3 UKK Institute for Health Promotion, 33501, Tampere, Finland; 4 Tampere School of Health Sciences, 33014 University of Tampere, Tampere, Finland; 5 Pediatric Research Centre, 33014 University of Tampere, and Tampere University Hospital, 33521, Tampere, Finland; 6 University of Eastern Finland, Kuopio University Hospital, 70211, Kuopio, Finland; 7 National Institute for Health and Welfare, 00271, Helsinki, Finland

**Keywords:** Pediatric, Intervention, Primary prevention, Childhood obesity, Diet therapy, Exercise, Follow-up, Controlled trial

## Abstract

**Background:**

Fetal conditions are known to be partly responsible for the child’s risk for obesity. Our pilot study aimed to determine the effect of gestational lifestyle counseling on the offspring weight gain until 4 years of age and to estimate power for future studies.

**Design and methods:**

First-time pregnant mothers participated in a controlled trial conducted in maternity health clinics during 2004 – 2006. The intervention included individual counseling on physical activity and diet, and an option to attend supervised group exercise sessions. The participant mothers (N = 109) received a follow-up questionnaire concerning 13 repeated growth measurements of their offspring. Response rate to the follow-up questionnaire was 66.1% (N = 72/109).

**Results:**

The increase of BMI z-score between 24–48 months was not significantly slower among the intervention group offspring (95% CI −0.025 to 0.009, p = 0.34) compared to control group. Z-scores for weight-for-length/height did not differ between groups when the period 0–48 months was analyzed (95% CI −0.010 to 0.014, p = 0.75).

**Conclusions:**

In this pilot study gestational lifestyle counseling did not significantly slow the weight gain of the offspring. Gestational intervention studies with at least 300 mothers per group are needed to confirm the possible effect on offspring’s risk for obesity.

**Trial registration:**

Current Controlled Trials ISRCTN21512277.

## Background

Childhood overweight and obesity have reached epidemic proportions in the past three decades [1-3]. Genetic susceptibility contributes to risk of obesity, but the present epidemic of obesity is mainly attributable to societal and environmental changes, with changes in lifestyle [4]. A large proportion of pregnant mothers are obese, and their offspring meet an obesinogenic environment prenatally. Mother’s prepregnancy BMI (body mass index), weight gain during pregnancy and glucose intolerance or gestational diabetes mellitus (GDM) seems to correlate with the offspring’s risk for subsequent overweight and obesity [5-9]. These prenatal influences on obesity risk are thought to be mediated by intrauterine programming via metabolic imprinting [9-12]. So far the exact mechanism which mediates the obesity risk on offspring during fetal life is unclear. There is some evidence that the increase in birthweight is not the only potential factor increasing offspring obesity risk [8,13,14]. Evidence from animal and some human studies suggests that epigenetic changes in metabolic control genes during fetal life have potential to affect offspring appetite and energy regulation as well as metabolism [15,16]. Poston et al. (2011) note in a recent consensus statement regarding obesity in pregnancy and long-term consequences on child health that “randomized controlled trials are urgently needed to evaluate the effect of nutritional and behavioral interventions in pregnancy on short- and long-term outcomes in mother and child” [9]. So far it has been unclear at what age the effect prenatal environment starts to influence offspring weight gain. In a recent work diabetes exposure *in utero* started to show after 27 months of age in offspring BMI development [17], and in the HAPO study there was no difference in offspring weight regarding maternal glucose level during pregnancy at the age of two years despite the significant effect of maternal glucose level on birthweight [7,18]. In several studies prepubertal offspring of mothers with GDM or maternal excess weight gain during pregnancy had a greater risk for overweight and adiposity [14,19-21].

Successful treatment of obesity is difficult even in childhood and high BMI in childhood increases risk of cardiovascular disease in adulthood [22]. Since excessive weight gain begins already during preschool years, preventive interventions should start early, before pregnancy, and include pregnancy and infancy [23]. So far only few such intervention studies targeting the preschool years have been published. In these studies some positive effects on child’s weight development have been found, but evidence of effective preventive means to reduce childhood obesity is still most insufficient [24-26]. To the best of our knowledge there are no published lifestyle counseling intervention trials targeting healthy pregnant women with follow-up of their offspring’s weight gain. In a randomized controlled trial by Gillman et al. (2010) they studied pregnant women with mild gestational diabetes and found that treatment of mild GDM reduced macrosomia at birth, but did not result in a lower BMI among the intervention offspring at the age of 4–5 years of age [27].

We hypothesized that healthier lifestyle during pregnancy could alter the intrauterine environment via mother’s more appropriate weight gain and also by improving mother’s glucose tolerance during pregnancy. Also, a healthier lifestyle adopted by the mother during the intervention could have positive effects on toddler diet and increase the time spent physically active. The aim of this study was to investigate whether intensified individual counseling on diet and physical activity targeting first time mothers during pregnancy affects offspring weight gain by the age of four years, and also to estimate the number of participants needed in future studies.

## Methods

### Study design

A controlled trial was conducted in six primary care maternity health clinics in Finland in the cities of Tampere and Hämeenlinna between the years 2004 and 2006. The study protocol was implemented during five visits to maternity health care clinics. The intervention study targeting mothers during their offspring’s first year has been reported earlier [28]. Feasibility of the study protocol and other details have been reported earlier [29-32].

The intervention study was conducted in six maternity care clinics, three of which volunteered to be intervention clinics and the remaining clinics were treated as control clinics. The allocation was performed at clinic level. The clinics were a convenience sample of the clinics in Tampere and Hämeenlinna as they were selected based on the clinics’ administrative personnel’s suggestion for suitable clinics. The nurses recruited pregnant women during their first visit to maternity health care. The eligibility of all potential participants was assessed and all eligible women were asked to participate in the study. The aim was to recruit at least 30 pregnant participants/clinic in the intervention and control clinics in August-October 2004.

For the original trial we used assumptions from previous literature resulting in 90% power and significance level α = 0.05, which suggested 82 women per group, in total 164 [33]. In addition, a conservative estimation of the sample size would be at least 1.5 fold compared to this calculation, because cluster randomization was applied. The estimated dropout rate (25%) was also taken into account in the sample size calculations. With these requirements, at least 300–350 women should be recruited for the study. However, the purpose of a pilot study was to test whether the study protocol was feasible and develop the protocol further before initiating the main study. The statistical significance of the results was not a priority in our pilot study and we aimed to recruit at least 60 pregnant women. Of these women, approximately 15 pregnant women were assumed to discontinue the study because of miscarriage, pregnancy complications or for other reasons.

### Participants

**Figure 1:**
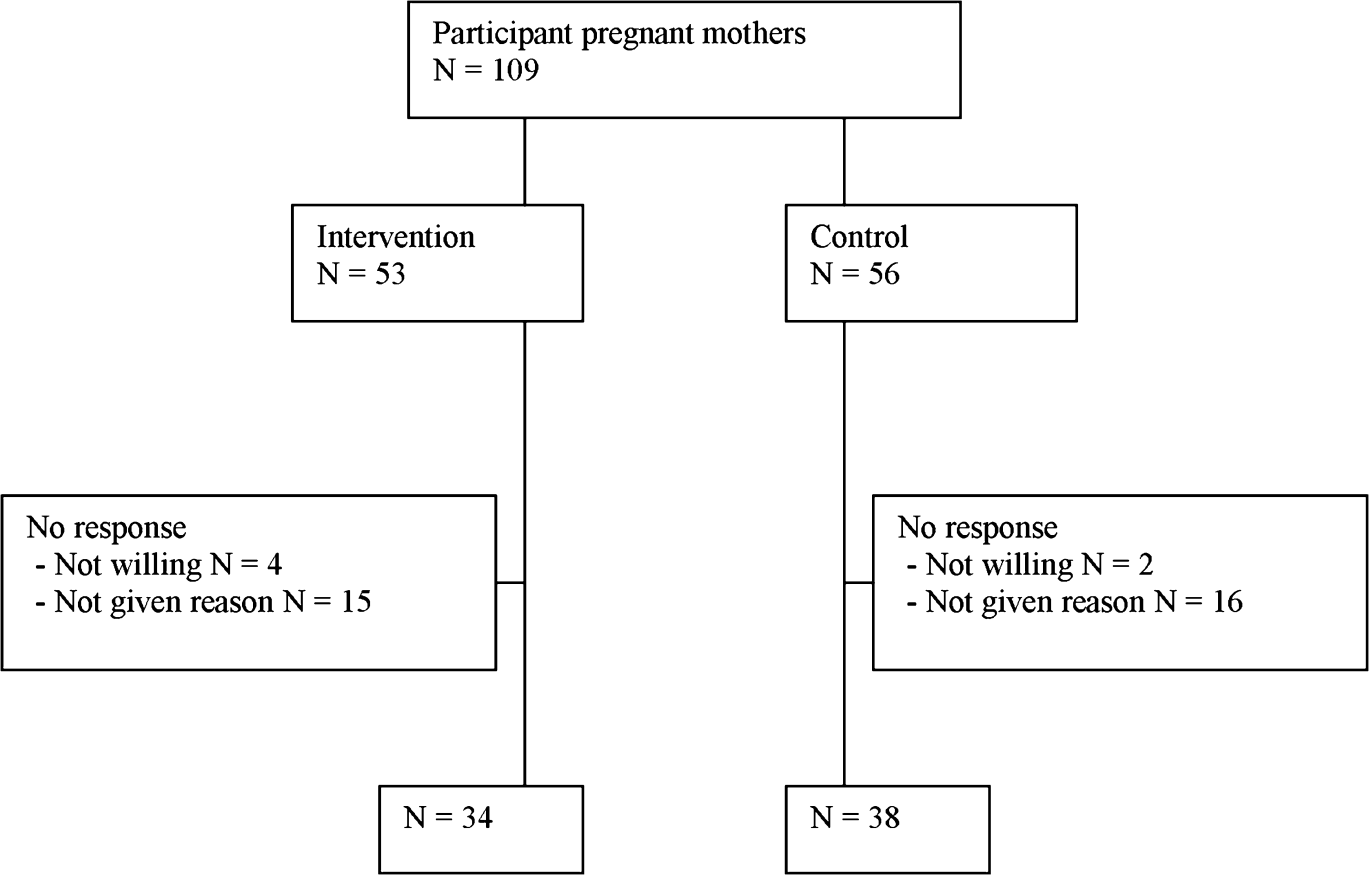
Original sample and the follow-up of the offspring, respondents and non-respondents.

The nurses recruited pregnant mothers with no previous deliveries (Figure 1). The exclusion criteria were age under 18 years, type 1 or type 2 diabetes mellitus (but not gestational diabetes mellitus), twin pregnancy, physical disability preventing exercising, otherwise problematic pregnancy (determined by a physician), substance abuse, treatment or clinical history for any psychiatric illness, inadequate language skills in Finnish and intention to change residence within three months. All participants provided written informed consent to participation.

### Intervention

The intervention included individual counseling on physical activity and diet at five routine visits to a maternity health care nurse starting at 8–9 weeks of gestation, and an option to attend supervised group exercise sessions once a week during pregnancy until 37 weeks’ gestation. The content of the intervention is described in greater detail elsewhere [29,31]. The purpose of the intervention was to promote leisure time physical activity and healthy dietary habits, thereby supporting participants’ to prevent excessive weight gain during pregnancy. In the control clinics, the nurses continued their usual counseling practices on physical activity and diet [31].

### Outcomes

In this study we analyzed the secondary outcome of the intervention study, namely the weight development of the offspring. The primary outcomes of the study have been reported earlier: Dietary outcomes were changes in meal patterns, overall intake of vegetables, fruit and berries, use of high-fiber bread and intake of high-sugar snacks. Physical activity outcomes were MET(metabolic equivalent) minutes [29,32]. The proportion of pregnant mothers exceeding the recommended level of gestational weight gain was also of interest as a primary outcome.

### Follow-up data collection

In 2010 mothers participating in the trial received a postal questionnaire on their offspring’s weight development. This questionnaire was chosen for data gathering as direct access to the child health clinic records would have entailed maternal permission, and mothers have the same information on their offspring’s growth as the child health clinic records. Finnish children attend child health care clinics several times in their first year and once a year thereafter. Children’s weight was measured to the nearest 0.1 kg on a standard electronic scale. Children under 2 years were measured in recumbent position and thereafter in standing position to the nearest millimeter with a standard stadiometer. A nurse enters the height and weight measurements in the child’s own health booklet for the mothers. The mothers entered these measurements in the postal questionnaire. The mothers were also asked whether their children had any long-term illnesses affecting growth (allergies or other chronic diseases), duration of breastfeeding and child’s age when starting solid foods.

### Statistical methods

The characteristics of the study participants were described using means and standard deviations or frequencies and proportions. The child’s size during follow-up was analysed using weight and length/height converted to BMI (weight (kg)/height (m^2^))-for-age and weight-for-length/height and their SDSs (z-scores) according to the recently updated Finnish growth reference [34-36]. The exact age of the child was used in all analyses.

Mixed-effects linear regression models were constructed to analyze the association of weight-for-length/height z-score and BMI z-score over time by group (intervention/control). Three-level mixed-effects models consisted of fixed effects (group, child’s age in months, nonlinear effects AgeInMonths^2^ and AgeInMonths^3^ and interaction between group and age) and random effects (measurements within child within centre). These models allow for a difference between groups at baseline, linear changes of z-score over time and the difference in improvement between groups, which can be viewed as the intervention effect (i.e. interaction term). A likelihood ratio test was used for model selection. The parameter estimates were presented with 95% confidence intervals (95% CI) and p-values. The goodness-of-fit of the models was evaluated visually by normal probability and residual plots and also tested by the normality of the residuals (Kolmogorov-Smirnov test). All analyses were performed using STATA software (version 12.0 for Windows), StataCorp LP, Texas, USA. Likelihood-ratio test was used in evaluating the change in model fit when including AgeInMonths^2^ and AgeInMonths^3^ in the models. We also performed analyses to compare mothers lost to follow-up and those who responded. Maternal prepregnancy BMI, education, marital and employment status and smoking before pregnancy were analyzed using Independent Samples *T*-test, Chi-Square Test or Mann–Whitney *U*-test. P-value for all these parameters between responders and non-responders were non-significant.

The study was approved by the Ethics Committee of the Pirkanmaa Hospital District.

## Results

Response rate to the follow-up questionnaire was 66.1% (N = 72/109). According to the loss-to-follow-up analysis, there were no statistically significant differences between responders and non-responders in age, BMI before pregnancy, employment status, education or smoking before pregnancy. Responses were missing from 37 women who received counseling during pregnancy (Figure 1). Women in the intervention group who responded to the questionnaire, were similar in age than the control women (mean age 28.7 vs. 29.1 years). There were no obese women with BMI ≥ 30 kg/m^2^ in the control group, whereas in the intervention group there were 3 women who were obese before pregnancy, and the proportion of normal weight (BMI < 25 kg/m2) mothers was higher among control group mothers (Table 1). There was no difference in the smoking status between the groups. The proportion of macrosomic infants was higher among the control mothers than among the intervention mothers (0.0% vs. 13.2%). Duration of breastfeeding or age when starting solid foods did not differ between the groups (Table 1).

**Table 1:** Baseline characteristics of the trial groups (mean ± sd or frequency and %)

	**Intervention**	**Control**	**p value**	**Missing**
N	34	38		
Age of the mother at delivery (years)	28.7 (4.2)	29.1 (3.6)	0.65 ^1^	-
Pre-pregnancy weight (kilograms)	64.2 (9.7)	61.7 (7.2)	0.22 ^1^	3, 1
Pre-pregnancy BMI (kg/m^2^)	23.3 (3.4)	22.2 (2.1)	0.12 ^1^	3, 1
Range	19.7 to 33.2	17.6 to 26.2		
Pre-pregnancy BMI			0.17 ^4^	3, 1
<25 (kg/m^2^)	24 (77.4%)	33 (89.2%)		
25-29.9	4 (12.9%)	4 (10.8%)		
30-	3 (9.7%)	-		
Gestational weight gain (kilograms)	13.6 (5.1)	14.1 (4.5)	0.69 ^1^	3, 1
Weight gain recommendations during pregnancy			0.59 ^3^	3, 1
Lower	11 (35.5%)	12 (32.4%)		
At the range of the recommendations	9 (29.0%)	15 (40.6%)		
Higher	11 (35.5%)	10 (27.0%)		
Education			0.25 ^3^	2, 0
Low	8 (25.0%)	11 (28.9%)		
Medium	7 (21.9%)	3 (7.9%)		
High	17 (53.1%)	24 (63.2%)		
Employed	28 (84.8%)	30 (78.9%)	0.52 ^3^	1, 0
Ever smokers	18 (54.5%)	18 (48.6%)	0.62 ^3^	1, 1
Smoking during pregnancy	0 (0.0%)	2 (5.3%)	0.50 ^4^	4, 0
Gestational age (days)	278.8 (10.7)	278.6 (8.4)	0.92 ^1^	1, 1
Sex of the child – boy	16 (47.1%)	18 (47.4%)	0.98 ^3^	-
Birthweight (grams)	3399 (313)	3388 (443)	0.91 ^1^	-
Proportion of children with SGA	2 (6.1%)	5 (13.2%)	0.32 ^3^	1, 0
Proportion of children with LGA	0 (0.0%)	0 (0.0%)	-	1, 0
Macrosomia, birthweight > 4,000 g	0 (0.0%)	5 (13.2%)	0.056 ^4^	-
Breastfeeding (no other nutrition) (months)	4.4 (1.6)	4.5 (1.7)	0.66 ^2^	0, 1
Partial breastfeeding (months)	7.2 (5.7)	7.5 (6.0)	0.95 ^2^	0, 1
Age of the child receiving solid foods (months)	5.0 (1.2)	5.0 (1.0)	0.90 ^1^	0, 1

*SGA* small for gestational age, *LGA* large for gestational age, ^1^ Independent Samples *T*-test, ^2^ Mann–Whitney *U*-test, ^3^ Chi-Square Test, ^4^ Fisher’s Exact Test.

**Figure 2:**
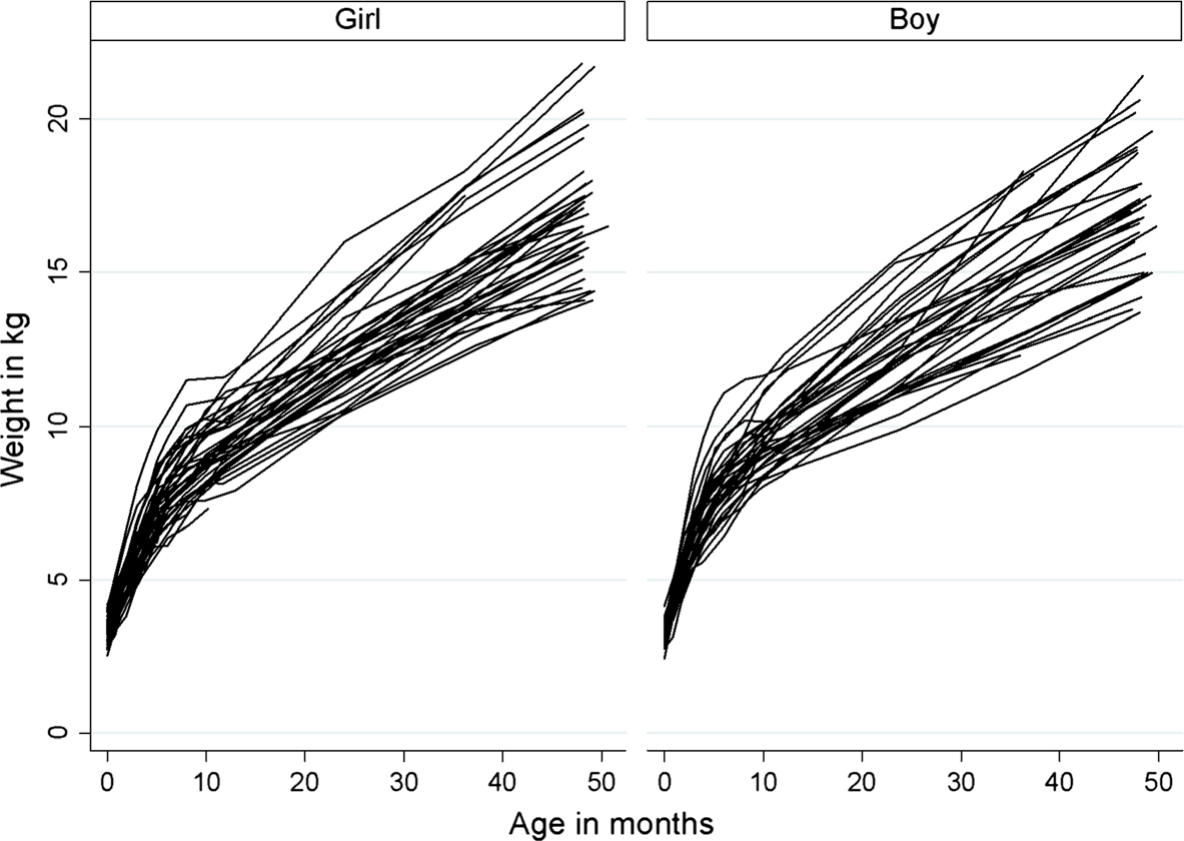
Growth trajectories by gender; exact age of the child and weight 0–48 months.

**Figure 3:**
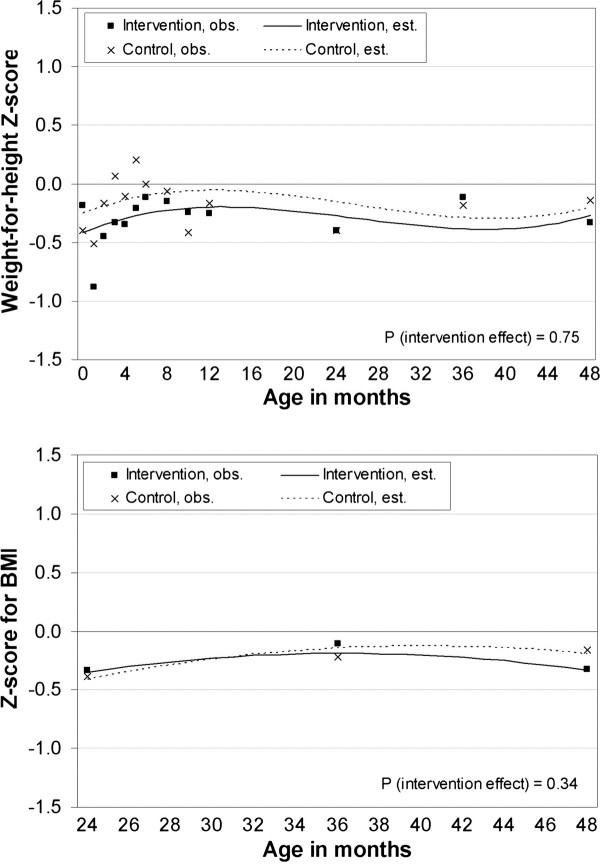
**Effect of intervention on offspring weight development.** Weight-for-length/height from birth to 48 months (upper) and BMI z-scores from 24–48 months (lower). P-values denote for the significance of intervention effects (interaction between group and child’s age at months). Non-linear model including group, age of the child, non-linear terms age^2^ and/or age^3^ and interaction between group and age of the child.

Observed weight trajectories were slightly wider among girls than among boys until the age of 48 months (Figure 2). Weight-for-length/height z-scores did not differ significantly between the intervention and control groups at birth (Table 1). Intra-cluster correlation for z-scores in the height/length-for-weight final model was 0.57 and for ZBMI 0.93. Weight-for-length/height z-score between birth and 48 months or BMI z-scores between 24 – 48 months did not differ statistically significantly between intervention and control group offspring (95% CI −0.01 to 0.01, p = 0.75 and 95% CI −0.03 to 0.01, p = 0.34) (Tablec 2, Figure 3). As possible confounders we analyzed maternal age and prepregnancy BMI, and gender of the child, but p-value for interaction did not become statistically significant in any of these. Mean weight-for-height z-score at 4 years of age did not differ significantly in groups: intervention group −0.333 and control group −0.141, p-value 0.47.

**Table 2:** Offspring weight development and confidence intervals

	**Coefficient**	**95% CI**	**p value**
**Weight-for-length/height z-** **score from 0 to 48 months of age**			
Group	−0.163	−0.563 to 0.237	0.42
Age	0.036	0.008 to 0.064	0.013
Age^2^	−0.002	−0.003 to −0.000	0.009
Age^3^	0.000	0.000 to 0.000	0.016
Group * Age	0.002	−0.010 to 0.014	0.75
**BMI z-score from 24 to 48months of age**			
Group	0.255	−0.611 to 1.121	0.56
Age	0.086	0.021 to 0.151	0.010
Age^2^	−0.001	−0.002 to −0.000	0.016
Group * Age	−0.008	−0.025 to 0.009	0.34

Estimates and 95% confidence intervals for z-scores for weight-for-length/height and body mass index. Results from separate multilevel mixed-effects linear regression models including group (intervention/control), age of the child and interaction between age of the child and group.

Ordinary sample size calculation assumes that all data points are independent. With a multi-level structure, the ordinary sample size estimates needs to be inflated by the design effect [1 + (*n* – 1)*ρ*, where *n* is the average cluster size and *ρ* is the estimated intra-cluster correlation coefficient (ICC). Sample size calculation proceeds by calculating the sample size for a naïve model, an ordinary model that assumes all observations are independent, and then inflating that sample size by multiplying it by the design effect, so that the sample size calculation applies to the multi-level model [37]. When comparing two groups of patients and 13 repeated measurements per child, the child is here considered as the cluster. Observed group means were −0.35 (intervention) vs. -0.15 (controls) and an intracluster coefficient = 0.57. Applying the design effect after calculating a sample size can be done in STATA (*sampclus)*. Thus we need 3,466 observations divided by the number of observations per child, 3466/13 = 266.6, or 267 child per group. When a drop-out rate of 20% is taken into account a sample size of at least 300 children in group is recommended in future studies.

## Discussion

The main finding of our study was that lifestyle counseling during pregnancy did not significantly slow weight gain among the offspring. A greater number of study participants and a longer follow-up period are needed in future studies. Based on the current differences between the groups, at least 300 children per group are needed in similar experimental studies.

Childhood obesity leads to substantially increased risk for type 2 diabetes and cardiovascular diseases [22,38]. There is growing evidence for the important role of early preventive efforts since the unfavorable health consequences of obesity begin already during childhood and the treatment of childhood obesity tends not to lead to permanent results [4,39]. The higher prepregnancy BMI, gestational weight gain and GDM of the mother,have been shown to increase the risk for childhood obesity [7,8,20,21,40]. Mother’s impaired glucose tolerance during pregnancy has been shown to increase offspring’s risk for obesity and adverse metabolic changes in several studies [13,19]. The mother’s glucose tolerance is influenced by diet and physical activity, as well as genetic factors and BMI. Our study was a controlled trial conducted in six maternity health clinics in primary care. The participants were first-time mothers without especially sought risk determinants for having overweight offspring. There were no statistically significant differences in factors known to affect offspring’s risk of obesity between the two groups: mother’s age before pregnancy, prepregnancy BMI, gestational weight gain, education, smoking during pregnancy and duration of breastfeeding. In the intervention clinics the mothers received intensified individual counseling on physical activity and diet, as well as an option to attend sessions of supervised group exercise once a week during pregnancy. The control group received conventional health care counseling. The intervention did not increase mother’s physical activity or prevent excess weight gain during pregnancy, but the intensified counseling increased pregnant mothers’ intake of fiber, vegetable and fruit (primary outcomes reported earlier) [29-32,41]. Thus this intervention could have an impact on the intrauterine environment via mother’s healthier diet. Gestational lifestyle intervention can also potentially influence offspring’s diet and time spent physically active via the healthier lifestyle adopted by their mothers. The role of parents is crucial in influencing lifestyle behavior among their offspring, and preschool age is an important period in the acquisition of food preferences and physical activity habits [23,42]. Previously published results from our data have shown a smaller proportion of macrosomic newborns in the intervention group than in control group [29]. Lawlor et al. (2011) showed in their recent study that the BMI of the offspring correlated with gestational weight gain if the mother was overweight or obese, but if the mother was of normal weight, the gestational weight gain had no correlation with offspring BMI, suggesting the role of intrauterine programming more clearly when the mother has high BMI [43]. In our study the majority of participating mothers were of normal weight, which may have influenced the results. In the study by Fraser et al. (2010) they found that any weight gain during the first 14 weeks of gestation was associated with increased offspring adiposity, but later in pregnancy only > 500 g/week weight gain increased offspring adiposity [40]. According to their result, the intervention targeting weight gain during pregnancy should start prior to conception rather than during the first trimester of pregnancy as in our study. The follow-up of the offspring of the HAPO study showed that maternal glucose at 28 weeks of gestation was not associated with offspring obesity at two years of age [18]. Crume et al. (2011) showed that intrauterine exposure to maternal gestational diabetes mellitus resulted in higher average BMI among the offspring only after 27 months of age and higher BMI growth starting at age 10 years and thus no differences in weight gain was seen in infancy or early childhood [17]. The follow-up period in our study probably should have been longer than four years to see the effect of intrauterine influences on offspring weight gain.

One weakness of our study was that the participants did not belong to risk groups such as mothers at risk for gestational diabetes or exclusively overweight/obese mothers. Another weakness was the relatively small number of participants in this pilot study. Moreover, the effect of lifestyle intervention would probably show more marked results in the reduction of offspring weight gain if the follow-up period of offspring growth had been longer than four years. One weakness of the study is lack of randomized design, since the clinics volunteered as intervention clinics due to the magnitude of the problems in their clients. Thus the intervention clinic mothers presumably had more adverse weight gain than the control mothers, which may have confounded the results in their offspring weight development as well.

The strengths of our study include a feasible counseling method, a controlled trial setting and reliable growth data based on repeated measurements by nurses in primary health care. We also utilized the recently updated growth data on Finnish children by using z-scores of weight-for-length/height and BMI-for-age described in that growth data. Our sample included healthy first-time mothers, thereby constituting a more homogeneous group than mothers with earlier deliveries. We were also able to take into account confounding factors on childhood growth, such as smoking and mothers’ prepregnancy BMI. A successful lifestyle intervention should create adequate motivation to change the lifestyle. Pregnancy is a suitable period to induce changes in lifestyle towards healthier, because pregnant mothers generally have good motivation to have a positive pregnancy outcome. Pregnant mothers have also regular contacts with health care nurses, and thus this kind of intervention is feasible. Our study was also integrated with primary health care follow-up of pregnancy. The intensified counseling helped pregnant mothers to increase the proportion of vegetables, fruit and fiber in their diet, as we have previously reported [25,28,41]. This change towards a lower glycemic index diet could improve mother’s glucose tolerance below gestational diabetes level as well as lower mother’s insulin levels. These metabolic changes may have beneficial sequelae in offspring weight gain by altering the intrauterine environment affecting the programming of offspring energy intake and metabolism.

## Conclusions

In our study the lifestyle counseling targeting pregnant mothers in maternity clinics did not significantly reduce the velocity of weight gain among the offspring by four years of age. To the best of our knowledge this study is the first published controlled lifestyle intervention trial targeting healthy mothers during pregnancy with follow-up of their offspring growth. Larger randomized controlled trials with at least 300 children per group targeting this crucial period with a longer follow-up period of the offspring growth are needed to ascertain whether pregnancy is a period when it is worth investing in lifestyle counseling interventions to combat the obesity epidemic.

## Abbreviations

BMI: Body mass index; GDM: Gestational diabetes mellitus; ICC: Intra-cluster correlation coefficient.

## Competing interests

The authors declare that they have no competing interests.

## Authors’ contributions

RL is the guarantor of the study. TM, PK and RL planned the follow-up questionnaire to the mothers. TM coded the data together with research assistant. AS produced BMI-for-age statistics and participated to the interpretation of the BMI-for-age results. JR performed the statistical analyses. All contributors participated in drafting of the manuscript and approved the final manuscript. All authors had full access to all of the data (including statistical reports and tables) in the study and can take responsibility for the integrity of the data and the accuracy of the data analysis.
